# Physical Basis for Juvenile Delinquency

**Published:** 1916-03

**Authors:** 


**Physical Basis for Juvenile Delinquency.** John A. Colliver, Los Angeles, Jour. of Sociologic Med., Dec. 1915. From probation court experience, the statement is made that 50% of juvenile cases are simply extensions of the normal impulse to play. It is also pointed out that a large number of violations of law would not have been so when we were boys—trespass, for example. This, of course, applies to grown persons also. The relief of criminal tendencies by removal of adenoids, circumcisions, attention to toxic and debilitated states is reported.

